# Broad Antiviral Activity of Carbohydrate-Binding Agents against the Four Serotypes of Dengue Virus in Monocyte-Derived Dendritic Cells

**DOI:** 10.1371/journal.pone.0021658

**Published:** 2011-06-30

**Authors:** Marijke M. F. Alen, Tine De Burghgraeve, Suzanne J. F. Kaptein, Jan Balzarini, Johan Neyts, Dominique Schols

**Affiliations:** Department of Microbiology and Immunology, Rega Institute for Medical Research, Katholieke Universiteit Leuven, Leuven, Belgium; Karolinska Institutet, Sweden

## Abstract

**Background:**

Dendritic cells (DC), present in the skin, are the first target cells of dengue virus (DENV). Dendritic cell-specific intercellular adhesion molecule 3-grabbing non-integrin (DC-SIGN) is present on DC and recognizes N-glycosylation sites on the E-glycoprotein of DENV. Thus, the DC-SIGN/E-glycoprotein interaction can be considered as an important target for inhibitors of viral replication. We evaluated various carbohydrate-binding agents (CBAs) against all four described serotypes of DENV replication in Raji/DC-SIGN^+^ cells and in monocyte-derived DC (MDDC).

**Methodology/Principal Findings:**

A dose-dependent anti-DENV activity of the CBAs *Hippeastrum hybrid* (HHA), *Galanthus nivalis* (GNA) and *Urtica dioica* (UDA), but not actinohivin (AH) was observed against all four DENV serotypes as analyzed by flow cytometry making use of anti-DENV antibodies. Remarkably, the potency of the CBAs against DENV in MDDC cultures was significantly higher (up to 100-fold) than in Raji/DC-SIGN^+^ cells. Pradimicin-S (PRM-S), a small-size non-peptidic CBA, exerted antiviral activity in MDDC but not in Raji/DC-SIGN^+^ cells. The CBAs act at an early step of DENV infection as they bind to the viral envelope of DENV and subsequently prevent virus attachment. Only weak antiviral activity of the CBAs was detected when administered after the virus attachment step. The CBAs were also able to completely prevent the cellular activation and differentiation process of MDDC induced upon DENV infection.

**Conclusions/Significance:**

The CBAs exerted broad spectrum antiviral activity against the four DENV serotypes, laboratory-adapted viruses and low passage clinical isolates, evaluated in Raji/DC-SIGN^+^ cells and in primary MDDC.

## Introduction

Dengue virus (DENV) belongs to the family of the *Flaviviridae* and is the most important emerging mosquito-borne virus in tropical and subtropical countries. According to the world health organization (WHO), two fifths of the world's population is at risk of getting infected with DENV (http://www.who.int/topics/dengue/en/). The virus can cause flu-like symptoms (dengue fever) that may progress to dengue hemorrhagic fever (DHF) and dengue shock syndrome (DSS). Dengue fever is characterized by a rapid onset of fever, headache, muscle and joint pain [1]. During a primary infection, most cases are self-limiting. There exist four genetically related serotypes of dengue virus. Infection with one serotype induces lifelong immunity to the homologous serotype. However, after infection with a second different serotype, the cross-reacting non-neutralizing antibodies against the first serotype will recognize the heterologous virus and enhance DENV access to Fc-receptor bearing cells [2]. This phenomenon is called antibody-dependent enhancement (ADE) and leads to a higher viremia, increased vascular permeability and a severe hemorrhagic disease [3], [4], [5], [6]. The first reported epidemic of DHF occurred in the Philippines in 1953 [7]. The past two decades, the global incidence of dengue fever has increased dramatically [8]. Reasons for the spread of dengue virus are the expansion of global population and travelling, deforestation, solid waste systems and poor vector control. The latter one is the only weapon against dengue virus, since there is no antiviral drug or vaccine available. Clinical studies with tetravalent chimeric dengue virus vaccines are ongoing [9], [10], [11].

Following the bite of an infected mosquito, immature dendritic cells (DC) in the skin are believed to be the first target cells during DENV infection [12]. Several cellular receptors for DENV have been proposed: heparan sulfate [13], LPS/CD14-associated binding proteins [14], heat shock protein (HSP) 90 and HSP70 [15] and the GRP78 liver receptor [16]. However, cell-surface C-type lectin DC-SIGN (CD209), mainly expressed by DC, is believed to be one of the most important receptors for DENV [17], [18], [19], [20]. DC-SIGN is a member of the calcium-dependent C-type lectin family and recognizes high-mannose glycans present on different pathogens such as human immunodeficiency virus (HIV) [21], hepatitis C virus (HCV) [22], ebola virus [23] and several bacteria, parasites and yeasts [24]. Many of these pathogens have developed strategies to manipulate DC-SIGN interaction to escape from an immune response [24]. Besides DC, macrophages play a key role in the immunopathogenesis of DENV infection. Recently, it was shown that the mannose receptor (MR; CD206) mediates DENV infection in macrophages by recognition of the glycoproteins on the viral envelope [25]. Monocyte-derived DC (MDDC), isolated from human donor blood, may not represent all *in vivo* DC subsets but they express both MR and DC-SIGN which make MDDC susceptible for DENV [17].

In most tissues, DC are in an immature state and they can capture the antigen because of their expression of attachment receptors, such as DC-SIGN. Following antigen capture in the periphery, DC maturate by upregulating their co-stimulatory molecules and migrate to lymphoid organs. Activated DC are stimulators of naive T-cells and they initiate production of cytokines and chemokines [26]. Inhibition of the initial interaction between DENV and DC could prevent an immune response and subsequently prevent cytokine release responsible for vascular leakage [27]. DC-SIGN could be a target for antiviral therapy by interrupting the viral entry process.

Here, we focus on the effect of various plant lectins, generally designated as carbohydrate-binding agents (CBAs). We showed previously that the CBAs have antiviral activity against DENV serotype 2 in Raji/DC-SIGN^+^ cells [20]. In the present study, we evaluated the antiviral activity of the CBAs in primary immature MDDC against the four different DENV serotypes and studied the kinetics of the antiviral activity of the CBAs during DENV infection.

## Results

### Preparation and characterization of MDDC

Human DC are the primary target cells for DENV infection. The DC used in our experiments were obtained through isolation of monocytes from buffy coats and further differentiated into MDDC in the presence of IL-4 and GM-CSF. Briefly, following centrifugation and aggregation, monocytes were cultured in media supplemented with IL-4 and GM-CSF for 5 days. The effect of IL-4 and GM-CSF treatment on the expression of several surface markers was analyzed by flow cytometry (Table 1). Monocytes efficiently differentiated into MDDC by IL-4 and GM-CSF, as evidenced by the significant decrease in CD14 expression and a significant increase in cell surface DC-SIGN (p<0.001). Also, other specific markers of DC, such as CD1a and CD11b, were significantly upregulated (p<0.05). The expression level of the DC maturation markers CD40 and CD83 were not markedly affected (p = 0.074 and p = 0.19 respectively), indicating that the MDDC used in our experiments are mainly in an immature cellular phase.

**Table 1:** Phenotypic analysis of monocytes and MDDC.

Cell type	CD14	CD1a	CD11b	CD40	CD80
Monocytes	95±1.3	0.22±0.17	66±7.2	0±0	0.12±0.01
MDDC	13±3.4	31±6.2	99±0.11	8.0±3.3	8.9±2.9
p-value	<0.001	<0.05	<0.05	0.074	<0.05

Cell type	CD83	CD86	DC-SIGN	MR	HLA-DR
Monocytes	1.9±1.2	35±8.5	0.53±0.22	59±11	88±4.8
MDDC	5.6±1.6	50±15	82±8.5	98±1.0	81±11
p-value	0.19	0.32	<0.001	<0.05	0.68

PBMCs were isolated from fresh donor blood buffy coats. After an aggregation step, the cells were cultured with or without 25 ng/ml IL-4 and 50 ng/ml GM-CSF for 5 days to derive MDDC or monocytes, respectively. Expression of several cell surface markers was analyzed flow cytometrically with specific PE-labeled mAbs. Data represent mean % positive cells ± standard error of the mean (SEM) from 5 different donors. The expression level of each marker was compared between monocytes and MDDC and the corresponding p-value was calculated with a paired *t*-test.

### Susceptibility of MDDC for DENV infection

**Figure 1:**
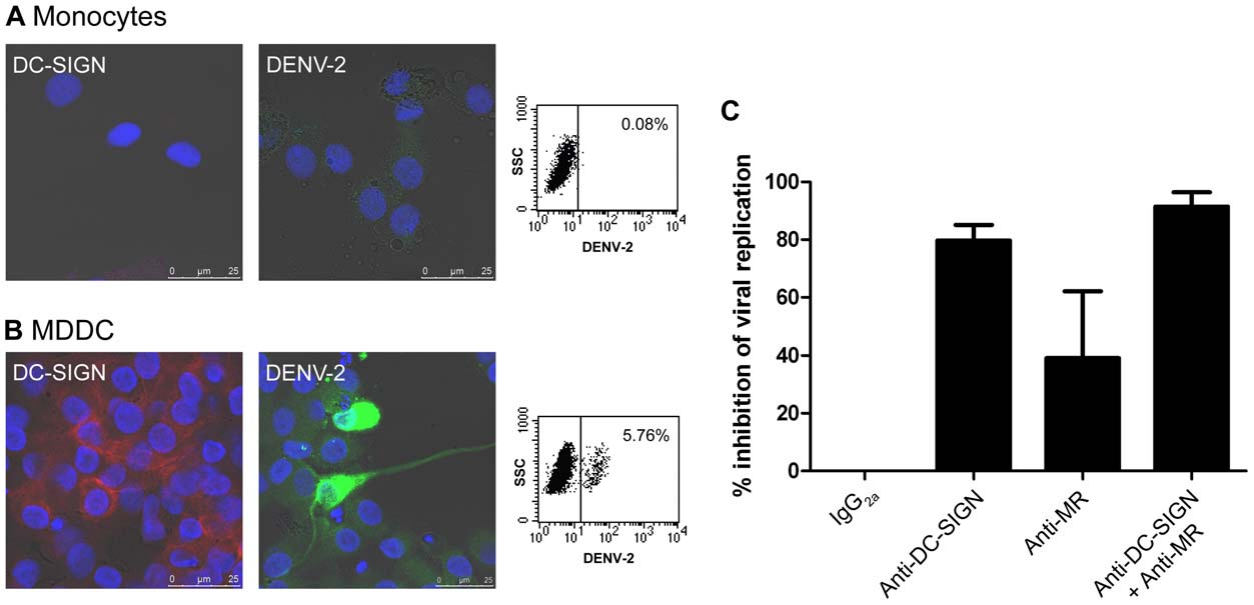
Infection of MDDC by DENV. Monocytes isolated from PBMCs were untreated (A) or treated with 25 ng/ml IL-4 and 50 ng/ml GM-CSF (B) for 5 days prior to DENV-2 infection. Two days after infection the cells were permeabilized and analyzed for DC-SIGN expression and DENV infection by confocal microscopy and flow cytometry. Uninfected cells were stained with a PE-labeled monoclonal DC-SIGN-antibody (red). Infected cells were stained with a mixture of antibodies recognizing DENV-2 E-protein and PrM protein (green). Nuclei were stained with DAPI (blue). Infected monocytes (A) and MDDC (B) were analyzed by flow cytometry to detect DENV-2 positive cells. The values indicated in each dot plot represent the % of DENV-2 positive cells. (C) MDDC were preincubated with 10 µg/ml of isotype control IgG_2a_, anti-DC-SIGN or anti-MR antibody for 30 minutes before DENV-2 infection. Viral replication was analyzed by flow cytometry. % Inhibition of viral replication ± SEM of 4 different blood donors is shown.

MDDC can be efficiently differentiated from monocytes by their expression of DC-SIGN. We examined the susceptibility of MDDC for DENV infection and the role of DC-SIGN in this infection process. Monocytes and immature MDDC were infected with DENV-2 and the infection was monitored by confocal microscopy and flow cytometry at day 2 post infection. Monocytes that are negative for DC-SIGN expression are very weakly susceptible for DENV-2 infection (Figure 1A). MDDC, with a high expression level of DC-SIGN, are readily infected with DENV-2 (Figure 1B). To investigate a possible role of DC-SIGN in DENV infection, we preincubated the MDDC with 10 µg/ml of a specific anti-DC-SIGN antibody 30 minutes prior to DENV-2 infection (Figure 1C). In parallel, an anti-MR antibody was included because MR was also described to interact with DENV glycoproteins [25] and is expressed by MDDC (Table 1). Flow cytometric quantification of viral infectivity revealed that treatment with the anti-DC-SIGN antibody had a marked inhibitory effect on DENV-2 infection (80% inhibition). Comparable results were obtained with the other serotypes of DENV (DENV-1, DENV-3 and DENV-4) (data not shown). Also, anti-MR antibodies diminished viral infection, although to a lesser extent. Combination of both anti-DC-SIGN and anti-MR antibodies prevented DENV-2 infection by >90% (Figure 1C).

### Antiviral activity profile of CBAs against the DENV serotypes

**Figure 2:**
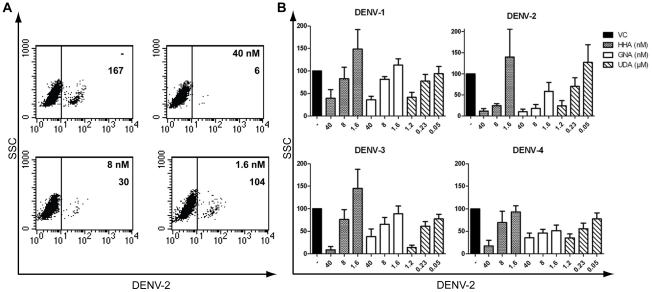
Dose-dependent antiviral activity of HHA, GNA and UDA in DENV-infected MDDC. (A) MDDC were infected with DENV-2 in the absence (−) or presence of dose-dependent concentrations of HHA. The number of DENV-2 positive cells was determined by flow cytometry using 5 µg/ml anti-DENV antibody recognizing the E-protein of DENV-2 (clone 3H5). In each plot, the number of DENV positive cells is indicated. (B) MDDC were infected with the four serotypes of DENV in the presence or absence of various concentrations of HHA, GNA and UDA. DENV infection was analyzed by flow cytometry using an anti-PrM antibody recognizing all four DENV serotypes (clone 2H2). % of infected cells compared to the positive virus control (VC) ± SEM of 4 to 12 different blood donors is shown.

**Figure 3:**
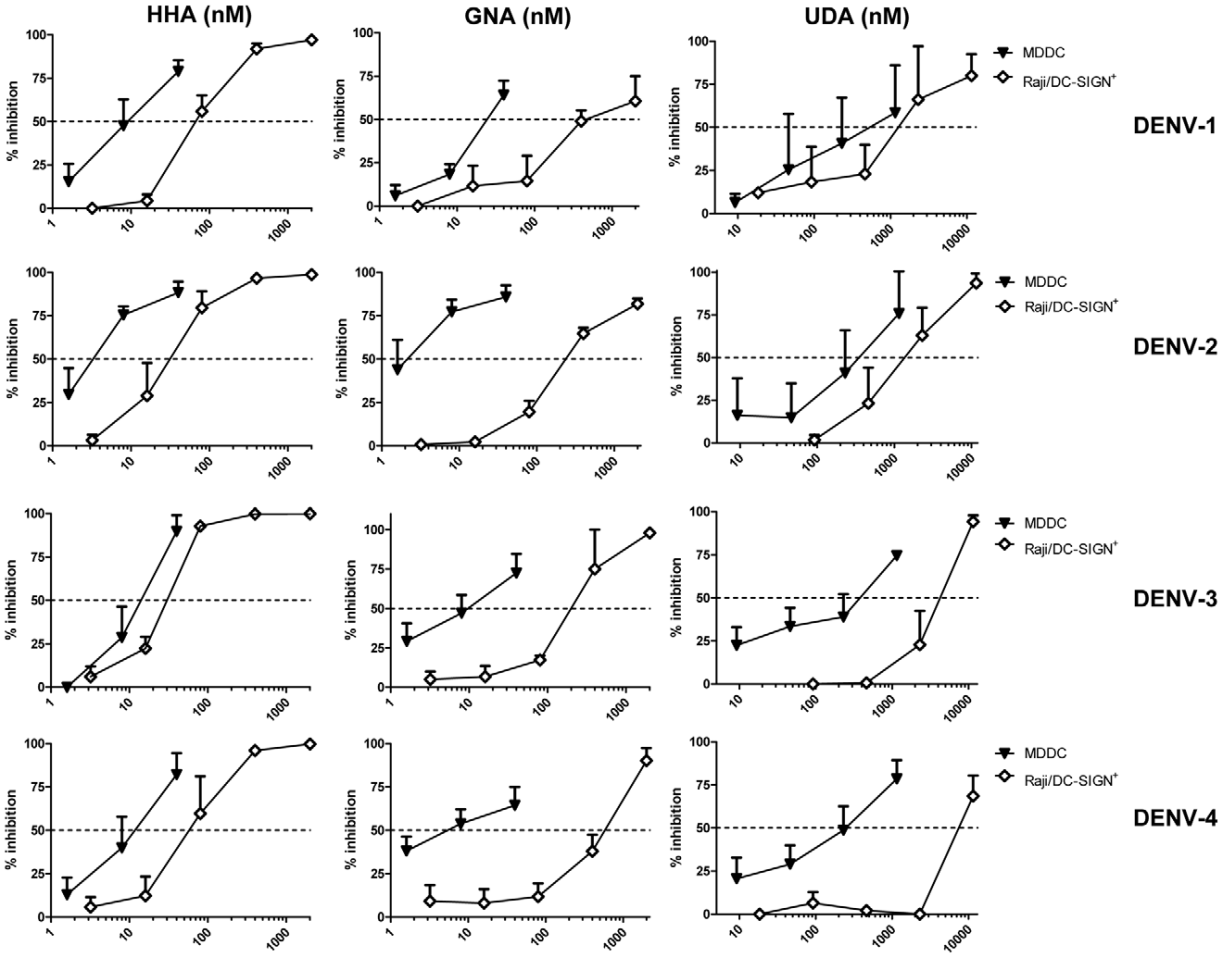
Antiviral activity of HHA, GNA and UDA in MDDC and in Raji/DC-SIGN^+^ cells. MDDC (▾) or Raji/DC-SIGN^+^ cells (□) were infected with DENV-1, DENV-2, DENV-3 or DENV-4. Cells were incubated with increasing concentrations of HHA, GNA, UDA and viral infectivity was quantified by flow cytometry using 5 µg/ml anti-DENV mAb (clone 2H2). Data represent the percentage of inhibition of viral replication relative to the positive control (DENV-infected cells). Each value represents the mean ± SEM of 3 to 8 independent experiments.

Thus, DC-SIGN and MR are crucial cellular viral receptors in DENV infection. Several plant lectins are known to interact with glycoproteins on the viral envelope. Previously, we demonstrated the antiviral activity of the mannose-specific lectins HHA, GNA and the GlcNAc-specific lectin UDA against DENV serotype 2 infection in Raji/DC-SIGN^+^ cells [20]. We now evaluated the ability of HHA, GNA and UDA to inhibit replication of all four DENV serotypes, i.e. laboratory-adapted DENV-2 and DENV-3 and low-passage clinical isolates DENV-1 and DENV-4, in primary immature MDDC. Viral infectivity in MDDC was quantified by means of flow cytometry at day 2 post infection, a time point when virus yield peaked in these cells (data not shown). MDDC were permeabilized and intracellularly stained with an anti-DENV antibody recognizing specifically the E-protein of DENV-2. Another antibody was used recognizing the PrM protein, detecting the four DENV serotypes. A dose-dependent inhibitory activity of HHA on DENV-2 infection in MDDC was observed by E-protein detection (Figure 2A). In addition, a dose-dependent inhibition by HHA, GNA and UDA against all four DENV serotypes in MDDC was observed (Figure 2B). The antiviral activity of all the CBAs evaluated was pronounced (nanomolar range). The plant lectins proved to be most active against DENV-2 infection (EC_50_ values of HHA, GNA and UDA of respectively 4.6, 3.8 and 480 nM). Next, we investigated whether the CBAs had similar broad antiviral activity in Raji/DC-SIGN^+^ cells. Under comparable experimental conditions, the three plant lectins had consistent antiviral activity against the four DENV serotypes in Raji/DC-SIGN^+^ cells (Table 2). In addition, comparison of the antiviral activity of HHA, GNA and UDA against the four serotypes of DENV between MDDC and Raji/DC-SIGN^+^ cells revealed a significant more pronounced antiviral activity of the CBAs in the MDDC. In fact, HHA, GNA and UDA were observed even to be up to 100-fold more active in MDDC than in Raji/DC-SIGN^+^ cells (Figure 3).

**Table 2:** Antiviral activity profile of various CBAs against the four serotypes of DENV.

		EC_50_ a			
CBA	Cell type	DENV-1	DENV-2	DENV-3	DENV-4
**HHA (nM)**	Raji/DC-SIGN	54±13	34±13	29±3.6	92±32
	MDDC	17±5.6	4.6±1.1	14±5.2	11±6
	p-value	<0.05	<0.05	0.05	0.099
**GNA (nM)**	Raji/DC-SIGN	280±102	240±32	170±60	560±174
	MDDC	22±4.2	3.8±1.5	11±4.4	5.6±1.4
	p-value	0.079	<0.05	0.092	<0.05
**UDA (µM)**	Raji/DC-SIGN	2.4±0.87	1.4±0.34	3.2±1.3	7.0±2.0
	MDDC	0.52±0.13	0.48±0.14	0.29±0.052	0.79±0.23
	p-value	0.12	<0.05	<0.001	<0.05

a EC_50_: 50% effective concentration, or drug concentration required to inhibit DENV infection in Raji/DC-SIGN^+^ cells and MDDC by 50% as measured by viral antigen expression. Values are the mean ± SEM of 3 to 10 independent experiments. EC_50_ values for each lectin and each DENV serotype was compared between Raji/DC-SIGN^+^ cells and MDDC. p-values were calculated with a *t*-test.

**Figure 4:**
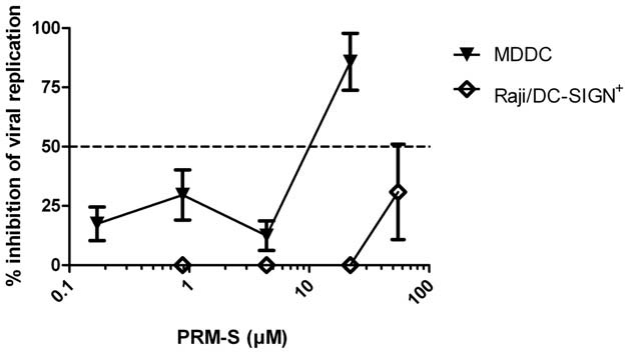
Dose-dependent antiviral activity of PRM-S in MDDC and in Raji/DC-SIGN^+^ cells. MDDC (▾) and Raji/DC-SIGN^+^ cells (□) were infected with DENV-2. Raji/DC-SIGN^+^ cells and MDDC were incubated with PRM-S at the start of the infection. The cells were collected and viral infectivity was quantified by flow cytometry using 5 µg/ml anti-DENV-2 mAb (clone 3H5). Data represent the mean % of inhibition of viral replication ± SEM of 3 independent experiments.

However, since plant lectins are expensive to produce and not orally bioavailable, the search for non-peptidic small molecules is necessary. PRM-S, a highly soluble non-peptidic small-size carbohydrate-binding antibiotic is a potential new lead compound in HIV therapy [28]. Since PRM-S efficiently prevents capture of HIV to DC-SIGN^+^ cells, we determined the antiviral activity of PRM-S against DENV-2 infection in Raji/DC-SIGN^+^ cell culture and in MDDC. PRM-S dose-dependently inhibited DENV-2 replication in MDDC (EC_50_: 11±2.9 µM) but had only a weak antiviral activity in Raji/DC-SIGN^+^ cells (EC_50_≥55 µM) (Figure 4).

Actinohivin (AH), a small prokaryotic peptidic lectin containing 114 amino acids, exhibits anti-HIV-1 activity by recognizing high-mannose-type glycans on the viral envelope [29]. Although DENV has high mannose-type glycans on the E-protein, we observed no antiviral activity of AH against DENV infection (EC_50_>1.6 µM).

Another small CBA is mAb 2G12 which is reported to be a broad neutralizing anti-HIV antibody [30]. The mAb 2G12 interacts with a well defined epitope on gp120 of HIV-1 (comprising at least three N-glycans) and this interaction is abrogated with soluble DC-SIGN [31]. The effect of mAb 2G12 on DENV infection in Raji/DC-SIGN^+^ cells and in MDDC was evaluated but no inhibitory activity of mAb 2G12 was observed (EC_50_>20 µg/ml).

### Effect of CBAs on virus-induced activation of MDDC

**Figure 5:**
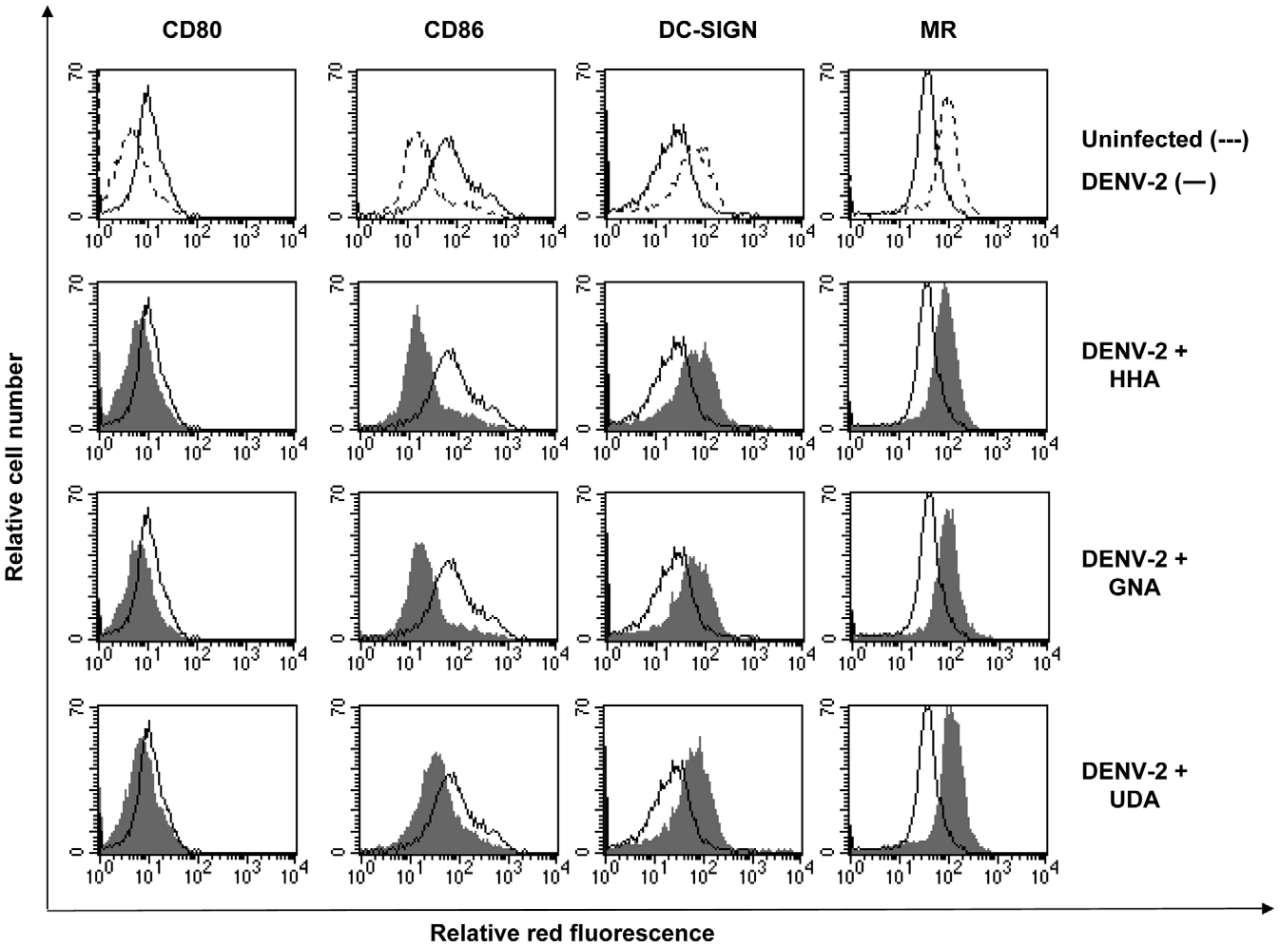
Differentiation process of MDDC induced by DENV-2 and inhibition of this process by CBAs. MDDC were infected with DENV-2 in the absence or presence of 400 nM HHA, GNA or 2.3 µM UDA. 5 days post infection, cell surface expression of CD80, CD86, DC-SIGN and MR were analyzed by flow cytometry with specific PE-labeled mAbs. Shown is the surface expression of the marker in uninfected cell culture (dashed line), untreated DENV-2-infected cell culture (full line) and the CBA-treated DENV-2-infected cell culture (full histogram). Data shown here are from 1 representative donor out of 4 different blood donors.

Viral infection of monocytic cells is known to evoke general activation of the cells. DC maturation and activation is characterized by upregulation of the costimulatory molecules CD80, CD86 and the DC specific marker CD83 [32], [33]. Therefore, the expression of several cell surface markers following infection with DENV-2 was analyzed by means of flow cytometry. It was observed that DENV-2 significantly upregulated CD80 (uninfected MDDC: 7.2±4.1% CD80^+^ versus infected MDDC: 35±10% CD80^+^, p<0.05) and CD86 expression (uninfected: 41±7.5% CD86^+^ versus infected: 94±1.6% CD86^+^, p<0.05) and significantly downregulated the expression of DC-SIGN (uninfected: 67±11% DC-SIGN^+^ versus infected: 34±12% DC-SIGN^+^, p<0.05). The expression of MR was downregulated, but not signficantly (p = 0.94) (Figure 5). DENV-2 infection did not alter the expression of CD83, CD40 and HLA-DR (data not shown). As a control, DENV-2 was inactivated by UV-irradiation and it was shown that UV-inactivated DENV-2 did not induce any significant cellular changes in MDDC as determined by CD80 (p = 0.87), CD86 (p = 0.25) and DC-SIGN (p = 0.74) expression. Also the plant lectin HHA as such did not induce any changes in the expression profile of MDDC (data not shown). These data indicate that all MDDC are affected by replicating DENV-2 infection, although only ∼5% of MDDC were positive for DENV-Ag expression. When 400 nM of HHA or GNA or 2.3 µM of UDA was added when MDDC were infected with DENV-2, the expression level of CD80, CD86 and DC-SIGN was almost identical to the expression level of the uninfected immature cell cultures (Figure 5). In conclusion, HHA, GNA and UDA not only efficiently inhibit DENV infection but also inhibit subsequent cellular activation levels of infected and uninfected MDDC induced by DENV-2 infection.

### Binding assays

**Figure 6:**
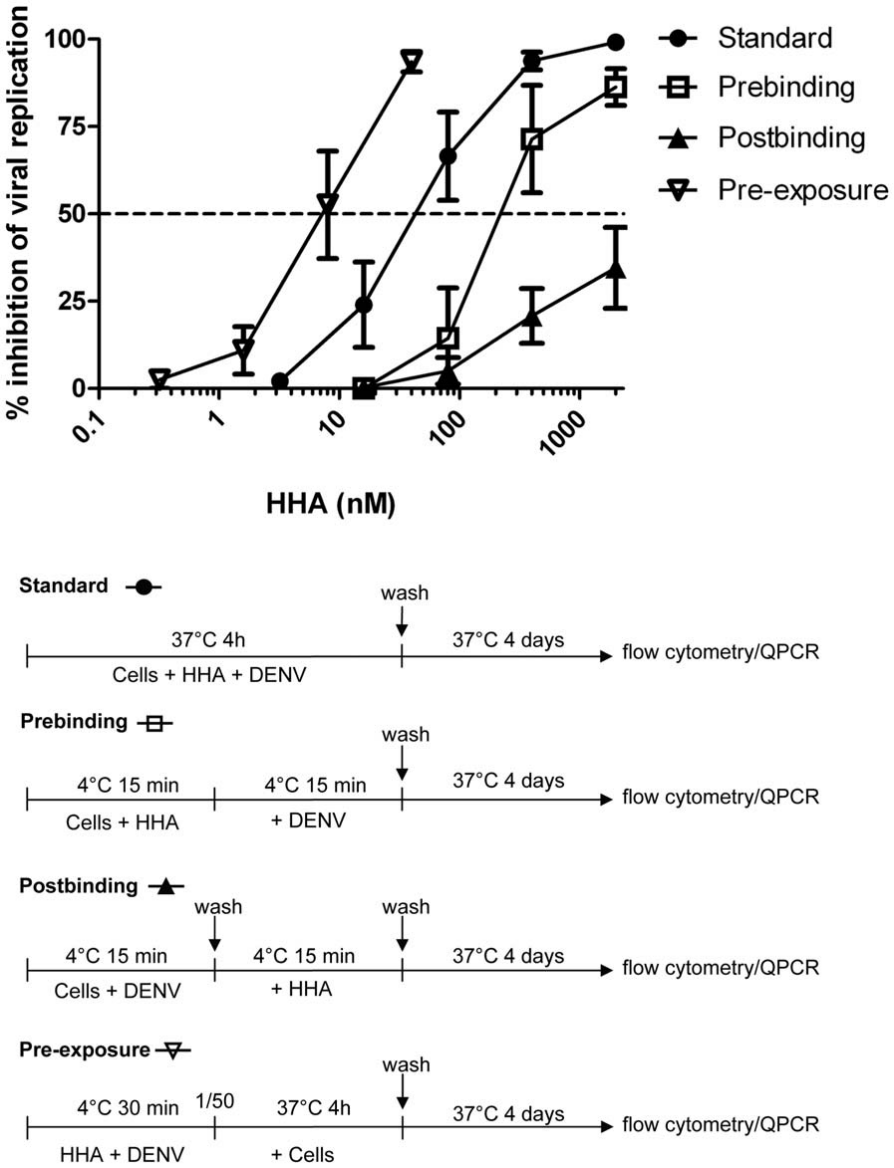
Antiviral assays with DENV-2 in Raji/DC-SIGN^+^ cells and HHA. Raji/DC-SIGN^+^ cells were infected with DENV-2 under four different experimental conditions. (•) Standard antiviral assay: cells were infected with DENV-2 together with HHA (2000-400-80-16-3.2 nM) and incubated at 37°C to allow internalization of the virus. (□) Prebinding assay: cells were pretreated with HHA (2000-400-80-16 nM) before binding to DENV-2. (▴) Postbinding assay: cells were infected with DENV-2 and after washing to remove unbound virus, HHA was added dose-dependently (2000-400-80-16 nM). (∇) Pre-exposure assay: cell-free DENV-2 was pre-exposed to HHA prior to exposure to Raji/DC-SIGN^+^ cells. Raji/DC-SIGN^+^ cells were infected with a 50-fold dilution of HHA-exposed DENV (final HHA concentration: 40-8-1.6-0.32 nM). For all four conditions, cells were washed twice to remove virus and/or compound and collected after 4 days. Viral infectivity was analyzed by flow cytometry. Data represent the mean % of inhibition of viral replication ± SEM of 3 independent experiments.

Experiments were set up to determine at which stage of infection the CBAs exert their inhibitory activity. Four different experimental conditions were used with Raji/DC-SIGN^+^ cells, DENV serotype 2 and HHA. In the first condition - i.e. the standard antiviral assay - HHA was present in the culture medium during the entire viral entry process. Briefly, cells were infected with DENV-2 in the presence of various concentrations of HHA and incubated for 4 hours at 37°C to allow internalization and replication of the virus. In the second condition - the prebinding assay - cells were first pretreated with HHA and then exposed to DENV-2 in the presence of HHA. In the third condition - the postbinding assay - cells were first incubated with DENV-2 for 15 minutes at 4°C, washed to remove unbound virus and treated with HHA. In the last condition - pre-exposure assay - cell-free concentrated DENV-2 was pre-exposed to HHA. This virus-HHA mixture was diluted 50-fold and exposed to Raji/DC-SIGN^+^ cells to investigate whether HHA interacts with DENV. In all four conditions, cells were finally washed to remove compound and/or virus and cultured in compound-free medium at 37°C for 4 days (Figure 6). A dose-dependent inhibition of DENV-2 infection was observed in all four conditions, as analyzed by flow cytometry (Figure 6) and confirmed by RT-PCR data. HHA proved most potent when cell-free virus was pre-exposed to HHA (pre-exposure assay: EC_50_: 8.0±6.8 nM) indicating that HHA efficiently interacts with the virus. The observation that the affinity of fluorescently labeled HHA to DENV-2-infected Raji/DC-SIGN^+^ cells is higher compared to uninfected cells also indicates that HHA selectively binds to DENV envelope proteins (data not shown). In comparison to the standard antiviral assay (EC_50_: 34±22 nM) a short pretreatment of the cells with HHA prior to virus infection resulted in a lower antiviral activity of the plant lectin (prebinding condition: EC_50_: 184±214 nM). When HHA was removed by washing the cells before DENV infection, no antiviral activity was observed, indicating also that HHA does not interact directly with DC-SIGN or other membrane proteins. In the postbinding condition, very weak if any activity of HHA was noted (EC_50_>2 µM), indicating clearly that HHA interferes with DENV attachment to the target cell. However, in this postbinding assay, treatment of the cells with HHA during the incubation period of 4 days at 37°C, resulted again in a significant inhibition of viral replication (EC_50_: 164±16 nM). Presumably, this is due to the fact that the virus undergoes multiple replication cycles and HHA can prevent the entry of newly synthesized virus particles. In conclusion, these data demonstrate that HHA binds to the envelope of the virus and not to cellular receptors such as DC-SIGN to prevent attachment and subsequent replication of the virus. Comparable results were obtained with other CBAs such as GNA in DENV-2 infection in Raji/DC-SIGN^+^ cells (data not shown).

## Discussion

Dendritic cells and macrophages are the cellular targets for DENV [12], [18], [34], [35], [36]. The four DENV serotypes used in our experiments were grown in the insect cell line C6/36 to mimic the first encounter of the DC with DENV. Thereby, infection of human primary MDDC with mosquito-derived DENV represents a good *in vitro* model to investigate the entry mechanism of DENV and the activity of specific antiviral compounds.

DENV-infected DC and macrophages play a key role in the immunopathogenesis of dengue hemorrhagic fever by the production of proinflammatory cytokines, chemokines, metalloproteinases and the induction of cell maturation [37], [38]. In most tissues, DC are in an immature state, unable to stimulate T-cells. They lack the expression of CD40 and CD86, the prerequisite for accessory signals for T-cell activation. However, immature DC are equipped with attachment receptors, such as DC-SIGN, to capture diverse pathogens [26]. We generated immature DC-SIGN expressing MDDC out of primary monocytes. Addition of IL-4 and GM-CSF to monocytes induces cell differentiation, DC-SIGN expression and enhances DENV susceptibility, consistent with other studies [25], [39]. DC-SIGN is nowadays hypothesized to be the main receptor for DENV, because it renders unsusceptible cells susceptible for DENV infection [20] and DC-SIGN is highly expressed in immature DC [17], [18], [19]. Another possible receptor for DENV is MR, expressed in immature DC and macrophages [25]. We confirm that DC-SIGN is an important receptor for DENV infection, because DC-SIGN-specific antibodies profoundly inhibit DENV infection of MDDC. Furthermore, the combination of anti-DC-SIGN and anti-MR antibodies was even more effective in inhibiting DENV infection. Yet complete inhibition of DENV infection was not achieved, indicating that other entry pathways are potentially involved. In the case of HIV, DC-SIGN is found to be an important attachment receptor on DC to capture HIV and transmit the virus to resting T-cells [21]. DC-SIGN-independent pathways for the transmission of HIV must exist, since anti-DC-SIGN mAbs and DC-SIGN small interfering RNA did not completely inhibit the transmission of HIV from DC to T-cells [40]. Two other receptors on DC reported to be responsible for HIV attachment are syndecan-3 (a member of the heparan sulfate proteoglycan family) [41] and the DC immunoreceptor [42]. Since DENV interacts with heparan sulfate, syndecan-3 may be a possible (co)-receptor on DC. It has been hypothesized that DENV needs DC-SIGN for attachment and enhancing infection of DC *in cis* and needs MR for internalization [25]. In fact, cells expressing mutant DC-SIGN, lacking the internalization domain, are still susceptible for DENV infection because DC-SIGN can capture the pathogen [19].

Interaction between DENV and DC-SIGN or MR is abrogated by deglycosylation of the DENV envelope and by EDTA or mannan [17], [25], indicating that the interaction is carbohydrate-dependent. DC-SIGN and MR have respectively 1 and 8 carbohydrate recognition domains (CRD) responsible for pathogen recognition [43], [44], [45]. Other pathogens recognized by DC-SIGN and MR are HIV, HCV and human cytomegalovirus (HCMV). These interactions are carbohydrate-dependent and are inhibited by various CBAs [46], [47], [48], [49]. In a previous report, we were the first to demonstrate the antiviral activity of the CBAs against DENV-2 in Raji/DC-SIGN^+^ cells and IL-4-treated monocytes [20]. In the present study, we studied the antiviral activity of these CBAs on the four DENV serotypes in primary MDDC, the most important target cells for DENV. A number of these CBAs proved about 100-fold more effective in inhibiting DENV infection in primary MDDC compared to the transfected Raji/DC-SIGN^+^ cell line.

We also demonstrated that the mannose binding lectin HHA prevents DENV-2 binding to the host cell and acts less efficiently in the postbinding stage. HHA interacts with DENV and not with DC-SIGN on the target cell. The potency of HHA to inhibit attachment of DENV to Raji/DC-SIGN^+^ cells is comparable to its inhibitory activity of the capture of HIV and HCV to Raji/DC-SIGN^+^ cells [47]. CBAs could thus be considered as unique prophylactic agents. However, plant lectins are not orally bioavailable, sensitive for proteolytic cleavage and expensive to produce [50], they provide novel insights into the entry mechanism of DENV in human primary cells. The search for non-peptidic small molecules with CBA-like activity is therefore warranted. PRM-S, a derivate of the antibiotic PRM-A, acts as a CBA in terms of glycan recognition and exerts antiviral activity against HIV and SIV [28]. The compound has high solubility and a high barrier for HIV resistance development. In MDDC, we observed a dose-dependent antiviral activity of PRM-S against DENV-2, comparable to the antiviral activity against HIV [28]. In contrast, PRM-S exerted only weak antiviral activity in Raji/DC-SIGN^+^ cells. Accordingly, the antiviral potency of the other CBAs, HHA, GNA and UDA was higher in primary MDDC than in Raji/DC-SIGN^+^ cells as well. This may be due to several cell-dependent specificities. First, Raji/DC-SIGN^+^ cells are more susceptible for DENV infection compared to MDDC (100% infected cells versus 5%, respectively) although the DC-SIGN expression level is comparable in the two cell-types. Although MDDC cultures contain a low amount of T-cells and B-cells, these cell types are not susceptible for DENV infection [51]. Second, the entry process of DENV in Raji/DC-SIGN^+^ cells (a transfected B-cell line) and in MDDC is fundamentally different. In Raji/DC-SIGN^+^ cells, the entry process is mainly dependent on DC-SIGN. This is in contrast to MDDC, where unidentified cofactors for infection or DC-SIGN-independent entry pathways of the virus may be present. Although, whatever entry pathway in MDDC is employed by the virus, DENV infection is efficiently inhibited by CBAs. This indicates that the DENV entry in human cells is carbohydrate-dependent and that CBAs also inhibit DC-SIGN-independent entry pathways in MDDC. Consequently, the observed antiviral activity of the CBAs in human primary MDDC may be considered more relevant than in artificial constructed cell lines such as the Raji/DC-SIGN^+^ cell line.

Another CBA with anti-HIV activity is AH, isolated from actinomycetes. We observed neither in Raji/DC-SIGN^+^ cells nor in MDDC antiviral activity of AH against DENV. This indicates that AH interacts rather specifically with high-mannose N-glycans on HIV-1 glycoprotein gp120 [29], [52], but not with DENV glycoprotein E.

The mAb 2G12 specifically recognizes a cluster of high-mannose-type oligosaccharides on HIV-1 gp120 [31]. mAb 2G12 could inhibit HIV binding to Raji/DC-SIGN^+^ cells [46] and could also bind to yeast glycoproteins [53]. We therefore assumed that mAb 2G12 could potentially recognize the E-protein of DENV, however no inhibitory effect on DENV was observed.

We can thus conclude that not all CBAs interact with all types of glycosylated enveloped viruses. The lectins HHA, GNA and UDA have a broad spectrum antiviral activity against HIV [46], SIV [54], HCV [47], HCMV [49], [55] and DENV but not against parainfluenza-3, vesicular stomatitis virus, respiratory syncytial virus or herpes simplex virus [47]. This may be because of differences in carbohydrate structures on the glycoproteins of the viral envelope of different viruses grown in different host cells. The glycosylation pattern in DENV differs from HIV because they replicate in mosquito cells and human cells, respectively. In vertebrate and invertebrate hosts the glycosylation process is fundamentally different [56], [57]. N-glycosylation in mammalian cells is often of the complex-type because a lot of different processing enzymes could add a diversity of monosaccharides. Glycans produced in insect cells are far less complex, because of less diversity in processing enzymes and usually contain more high-mannose and pauci-mannose-type glycans.

When DENV is captured by DC, a maturation and activation process occurs. DC require downregulation of C-type lectin receptors [58], upregulation of costimulatory molecules, chemokine receptors and enhancement of their APC function to migrate to the nodal T-cell areas and activate the immune system [59]. Cytokines implicated in vascular leakage are produced, the complement system becomes activated and virus-induced antibodies can cause DHF via binding to Fc-receptors. Several research groups demonstrated maturation of DC induced by DENV infection [32], [33]. Some groups made segregation in the DC population after DENV infection, the infected DC and the uninfected bystander cells. They found that bystander cells, in contrast to infected DC, upregulate the cell surface expression of costimulatory molecules, HLA and maturation molecules. This activation is induced by TNF-α and IFN-α secreted by DENV-infected DC [27], [60], [61]. We observed an upregulation of the costimulatory molecules CD80 and CD86 and a downregulation of DC-SIGN and MR on the total (uninfected and infected) DC population following DENV infection. This could indicate that the DC are activated and can interact with naive T-cells and subsequently activate the immune system resulting in increased vascular permeability and fever. When we examined the effect of the CBAs on the expression level of the cell surface markers of the total DC population, we are able to inhibit the activation of all DC caused by DENV and keeping the DC in an immature state. Furthermore, DC do not express costimulatory molecules and can not interact nor activate T-cells. An approach to inhibit DENV-induced activation of DC may prevent the immunopathological component of DENV disease. Immunoglobulin G was previously shown to inhibit the differentiation and maturation of DC *in vitro* indicating that the DC activation process is an important target for controlling immune responses in several diseases [62].

In conclusion, we observed broad spectrum antiviral activity of HHA, GNA and UDA against all four serotypes of DENV, laboratory-adapted strains and low-passage clinical isolates, evaluated in primary MDDC. The DENV MDDC infection model mimicks more closely a primary infection of DC *in vivo* than other infection models. CBAs act by binding to DENV glycoproteins and subsequently interrupt the interaction between DENV and DC-SIGN. Our data provide more insight into the mechanism of action of the CBAs in MDDC and indicate the relevance of the carbohydrate-dependent entry pathway of DENV in primary human cells. It is important to further develop therapeutic concepts that may prevent DENV-induced diseases. Small-size non-peptidic analogues, such as PRM-S, should be further pursuit for this purpose.

## Materials and Methods

### Cell lines and viruses

All cell cultures were maintained at 37°C in a humidified, CO_2_-controlled atmosphere, except for C6/36 mosquito cells (isolated from *Aedes albopictus*; ATCC CRL-1660), which were maintained at 28°C in the absence of CO_2_. C6/36 cells were grown in Minimum Eagle's Medium (MEM) (Invitrogen, Merelbeke, Belgium) supplemented with 10% fetal bovine serum (FBS) (Hyclone, Perbio Science, Aalst, Belgium), 0.01 M HEPES buffer (Invitrogen), non-essential amino acids (Invitrogen), 2 mM L-glutamine (Invitrogen), 100 units/ml penicillin and 100 units/ml streptomycin (Invitrogen). Raji/DC-SIGN^+^ cells were constructed by Geijtenbeeck et al. [21] and were kindly provided by Dr. L. Burleigh (Pasteur Institute, Paris, France). Raji/DC-SIGN^+^ cells were cultivated in RPMI-1640 medium supplemented with 10% FBS and 2 mM L-glutamine. African green monkey kidney cells (Vero-B cells; ATCC CCL-81) were grown in MEM (Invitrogen,) supplemented with 10% FBS, 2 mM L-glutamine and 0.075% sodium bicarbonate (Invitrogen).

Dengue virus (DENV) serotype 2 laboratory-adapted New Guinea C (NGC) strain was kindly provided by Dr. V. Deubel (Institut Pasteur, Paris, France). Low-passage clinical isolate DENV serotype 1 Djibouti strain D1/H/IMTSSA/98/606 (Genbank Accession Number AF298808), laboratory-adapted DENV serotype 3 strain H87 (prototype) (Genbank Accession Number M93130) and low-passage clinical isolate DENV serotype 4 strain Dak HD 34 460 (no complete sequence available, only partial unpublished sequences) were kindly provided by Dr. X. de Lamballerie (Université de la Méditerranée, Marseille, France). All four DENV serotypes were propagated in C6/36 cells. Supernatant containing virus was harvested 5 days post infection and stored at −80°C. Titer of DENV was determined in Vero-B cells by validation of cytophatic effects to obtain the cell culture infective dose infecting 50% of the cells (CCID_50_)/ml value. In some experiments, DENV-2 virus stock was inactivated by ultraviolet (UV)-irradiation from a 30 W germicidal lamp at a distance of 10 cm for 15 minutes at room temperature. UV-inactivated DENV-2 was not able to infect Raji/DC-SIGN^+^ cells indicating the absence of replicating virus (data not shown).

### Isolation and differentiation of MDDC from human PBMCs

Buffy coat preparations from healthy donors were obtained from the Blood Bank in Leuven, Belgium. Human peripheral blood mononuclear cells (PBMCs) were first isolated by density gradient centrifugation over Lymphoprep (Nycomed, Oslo, Norway). PBMCs were gently rotated at 4°C to form aggregates of monocytes. After sedimentation of the monocytes, the pellet was grown in RPMI culture medium supplemented with or without 25 ng/ml IL-4 and 50 ng/ml GM-CSF (Peprotech, London, United Kingdom). After 5 days, IL-4 and GM-CSF differentiated monocytes into immature MDDC as analyzed by various cellular markers by flow cytometry (Table 1).

### Test agents

The mannose-specific plant lectins from *Hippeastrum hybrid* (HHA) (50 kDa), *Galanthus nivalis* (GNA) (50 kDa) and the N-acetylglucosamine (GlcNAc)-specific plant lectin from *Urtica dioica* (UDA) (8.7 kDa) were derived and purified from these plants as described previously [63], [64]. Pradimicin-S (PRM-S, 910 Da) was isolated from *Actinomadura* sp. TP-A0020 as described previously [65]. Actinohivin (AH) (12.5 kDa) was prepared from a culture broth of the actinomycete strain L. albida K97-0003 as described previously [66], [52]. The 2G12 mAb was purchased from Polymun Scientific (Vienna, Austria). Purified DC-SIGN (clone 120612) and MR (clone 19.2) antibody were purchased from R&D Systems (Minneapolis, MN, USA) and BD (BD Biosciences, Erembodegem, Belgium), respectively. Purified mouse IgG_2a_ isotype control antibody (clone MOPC-173) was purchased from BD Biosciences.

### Antiviral assays

Cells were seeded in flat-bottom polystyrene plates (Iwaki, International Medical Products, Belgium) and infected with an inoculum of DENV that caused 100% infected Raji/DC-SIGN^+^ cells analyzed by flow cytometry at day 4 post infection (∼100 CCID_50_/ml of each DENV serotype). Raji/DC-SIGN^+^ cells (0.5 10^6^ cells/well), monocytes and MDDC (1.5 10^6^ cells/well) were infected with the four different DENV serotypes in the absence or presence of compound for 4 hours at 37°C. The cells were washed twice with medium to remove excessive virus and compound and were further incubated at 37°C in fresh culture medium. Monocytes, MDDC and Raji/DC-SIGN^+^ cells were used at day 2 or 4 post infection, respectively, to detect DENV antigen by flow cytometry, RT-PCR or by confocal microscopy.

### Binding assays

Raji/DC-SIGN^+^ cells were infected with DENV-2 under four different experimental conditions: the standard antiviral assay as described above, a prebinding assay, a postbinding assay and a pre-exposure assay (Figure 6).

Prebinding assay: Raji/DC-SIGN^+^ cells were preincubated with various concentrations of HHA (2000-400-80-16 nM) for 15 minutes at 4°C. Then the cells were incubated with DENV-2 for 15 minutes at 4°C, a temperature that only allows virus binding to occur. After the incubation period, cells were washed excessively to remove unbound virus and were further incubated for 4 days at 37°C to allow viral internalization and replication.

Postbinding assay: Raji/DC-SIGN^+^ cells were preincubated with DENV-2 for 15 minutes at 4°C. Cells were washed with medium to remove unbound virus. The cells were then treated with different concentrations of HHA (2000-400-80-16 nM) and incubated at 4°C for 15 minutes. After the incubation period the cells were washed thoroughly with medium and the cells were further cultured in compound-free medium for 4 days at 37°C.

Pre-exposure assay: concentrated cell-free DENV-2 (5000 CCID_50_/ml) was pre-exposed to HHA (2000-400-80-16 nM) for 30 min at 4°C. Then, the drug-exposed virus was diluted (50-fold) in a way that the same amount of virus as in the standard antiviral assay was exposed to the cells (final lectin concentration 40-8-1.6-0.32 nM). The cells were incubated for 4 hours at 37°C and after 2 washing steps, cells were further cultured for 4 days at 37°C in the absence of product. Then, the cells were collected to analyze DENV antigen expression by flow cytometry and supernatants were collected to extract the RNA and quantify the amount of viral RNA by RT-PCR.

### Flow cytometry analysis

The expression of surface molecules on monocytes and MDDC was analyzed 5 days after isolation. Floating cells were collected and remaining adherent cells were scraped off using a cell scraper (BD Biosciences). Cells were washed with phosphate buffered saline (PBS) supplemented with 2% FBS and stained with phycoerythrin (PE)-labeled monoclonal antibodies recognizing CD14, CD1a, CD11b, CD40, CD80, CD83, CD86, MR, HLA-DR (BD Biosciences) and DC-SIGN (clone 120507, R&D Systems) for 30 minutes at room temperature. As a negative control for background staining, cells were stained in parallel with simultest control γ_1_/γ_2a_ (BD Biosciences). After a washing step with PBS, cells were fixed with 1% formaldehyde.

Raji/DC-SIGN^+^ cells were infected with the four different DENV serotypes and analyzed by flow cytometry 4 days post infection, as described previously [20]. Briefly, Raji/DC-SIGN^+^ cells were collected, washed with PBS supplemented with 2% FBS and stained with 5 µg/ml monoclonal anti-dengue virus type 2 antibody, specific to the E-glycoprotein of DENV type 2 NGC (clone 3H5, Chemicon International/Millipore, Billerica, MA) or with an antibody recognizing the premembrane (PrM) protein of all DENV serotypes (clone 2H2, Millipore). After an incubation period of 30 minutes at room temperature, cells were washed with PBS and incubated with secondary PE-conjugated goat F(ab′)_2_ anti-mouse antibody (1 µg/ml, Caltag Invitrogen Carlsbad, CA, USA) for 30 minutes at room temperature. Finally the cells were washed with PBS and fixed with 1% formaldehyde.

MDDC were infected with DENV and incubated for 2 days. After collecting the cells, cells were washed with PBS, fixed and permeabilized using the Cytofix/Cytoperm Kit (BD Biosciences) according to the manufacturer's instructions. Briefly, cells were fixed and permeabilized with cytofix/cytoperm buffer at 4°C for 20 minutes. After washing the cells with perm/wash buffer the permeabilized cells were incubated with 5 µg/ml anti-DENV Ab (clone 3H5 for serotype 2 or clone 2H2 for the other serotypes) for 30 minutes at 4°C. Following a washing step, the secondary PE-conjugated goat F(ab′)_2_ anti-mouse Ab (Caltag Invitrogen) was added and incubated at 4°C. As a control for unspecific background staining, Raji/DC-SIGN^+^ cells and MDDC were stained in parallel with secondary antibody only. To avoid any crossreactions, MDDC preincubated with anti-DC-SIGN Ab were stained with 5 µg/ml anti-DENV Ab directly labeled with alexa fluor 488 IgG labeling kit (Zenon, Invitrogen) according to the manufacturer's instructions. The stained cells were washed and analyzed by flow cytometry with a FACSCalibur (BD Biosciences, San Jose, CA). Data were acquired and analyzed with CellQuest software (BD Biosciences). The mean fluorescence of intensity (MFI) of the background staining was subtracted from the MFI of each sample to obtain the number of DENV-infected cells.

#### RNA extraction and Real Time (RT)-PCR

Total RNA was extracted from 150 µl cell culture supernatant using the Nucleospin RNA Virus Kit according to the manufacturer's instructions (Macherey-Nagel, Düren, Germany). RT-PCR was performed as described previously [20]. Briefly, the sequences of the forward (5′-TCGGAGCCGGAGTTTACAAA-3′ position 4628–4647) and reverse (5′-TCTTAACGTCCGCCATGAT-3′, position 4722–4741) Taqman primers were selected from non-structural gene 3 (NS3) of DENV NGC using Primer Express software (version 2.0, Applied Biosystems, Lennik, Belgium). The probe was selected between the primers and is fluorescently labeled with 6-carboxyfluorescein (FAM) at the 5′ end as the reporter dye and with a quencher at the 3′ end. The quencher is a minor groove binder (MGB) (5′-FAM-ATTCCACACAATGTGGCA-MGB-3′, position 4656–4674). The nucleotide sequence and position of the primers and probes were obtained from the nucleotide sequence of DENV 2 NGC (Genbank accession no. M29095) [67]. One step RT-PCR was performed in a 25 µl reaction mixture containing 12.5 µl One-Step Reverse Transcriptase qPCR Master Mix (Eurogentec, Seraing, Belgium), 900 nM forward primer, 900 nM reverse primer, 200 nM probe and 100 ng sample RNA. RT-PCR was performed under the following conditions: reverse transcription at 48°C for 30 min, initial denaturation at 95°C for 10 min, followed by 40 cycles of denaturation at 95°C for 15 s, annealing and extension at 60°C for 1 min. RT-PCR was performed using the ABI 7500 Fast Real-Time PCR System (Applied Biosystems, Branchburg, New Jersey, USA) and data were analyzed with ABI PRISM 7500 SDS software (version 1.3.1, Applied Biosystems). Standard curves were made of dengue virus plasmid with known concentrations to calculate the absolute quantification of infection.

#### Confocal microscopy

Isolated monocytes were seeded in coverslips to allow adherence. Monocytes were grown in RPMI culture medium. To generate MDDC, monocytes were incubated with IL-4 and GM-CSF. After 5 days, monocytes and MDDC were infected with DENV-2. 2 days post infection, cells were fixed with 3.7% formaldehyde for 15 minutes at room temperature and permeabilized with 0.1% Triton X-100 for 10 minutes at room temperature. After several washing steps, cells were incubated with 2% bovine serum albumine (BSA) (Sigma-Aldrich, St. Louis, MO, USA) in PBS to block Fc-receptors. Next, cells were stained with a mixture of anti-DENV Ab (clone 2H2 and clone 3H5) followed by incubation with a secondary antibody goat-anti-mouse IgG Alexa fluor 488 (Invitrogen). The coverslips were mounted with prolong gold antifade reagent and DAPI (Invitrogen) to stain the nucleus and incubated at 4°C until the cells were processed for microscopic analysis. Images were collected with a Leica TCS SP5 laser scanning confocal microscope (Leica Microsystems, Mannheim, Germany) equipped with an AOBS, using a HCX PL APO 63.0× (NA:1.40) oil immersion lens. The different fluorochromes were detected sequentially using excitation lines of 405 nm (DAPI), 488 nm (Alexa fluor 488) or 561 nm (PE). Emission was detected between 410–475 nm; 493–575 nm and 566–675 nm, respectively.

#### Statistical analysis

Statistical analysis performed on the results included the calculation of the mean, SEM and p-values by use of a paired or unpaired *t*-test. The significance level was set at p<0.05. Statistical analysis was performed with GraphPad Prism statistical software (GraphPad Software, Inc., San Diego, CA).
